# Westminster Hospital

**Published:** 1914-01-17

**Authors:** J. Wolfe Barry

**Affiliations:** Chairman of the House Committee of Westminster Hospital. Westminster


### Westminster Hospital.

SIR,—Will you kindly permit me to inform your readers that the negotiations for the acquisition of Westminster Hospital by the Dominion of Canada which have been under discussion for some months past have recently been terminated by the decision of the Canadian Government that, after full consideration, it would not acquire any site for a Dominion building in London in the immediate future? The site of the hospital is therefore available for other purposes, as the policy of the governors of moving the hospital to a position more commodious and convenient for hospital purposes on the completion of a contract for the purchase of the existing site remains in full force.—I am, Sir, your obedient servant,

J. WOLFE BARRY,
Chairman of the House Committee
of Westminster Hospital.

Westminster, January 10.

NOTE.—Owing to the pressure on our space other letters have had to be held over.—ED.

